# A survey of feeding, activity, supplement use and energy consumption in North American agility dogs

**DOI:** 10.1017/jns.2017.44

**Published:** 2017-08-29

**Authors:** Gina K. Dinallo, Jennifer A. Poplarski, Gretchen M. Van Deventer, Laura A. Eirmann, Joseph J. Wakshlag

**Affiliations:** 1 Department of Clinical Sciences, Cornell University College of Veterinary Medicine, Ithaca, NY 14853, USA; 2 Nestlé Purina Corporation, Professional Engagement, St Louis, MO, USA

**Keywords:** Agility, Energy, Diet, Supplements, Conditioning, BCS, body condition score, USDAA, United States Dog Agility Association

## Abstract

A survey was designed and administered at eighteen agility competitions across the Northeast and Midwest USA in 2015 to obtain information regarding competition level, training, feeding practices, owner-reported weight, body condition score (BCS) and supplement use. Average energy intake per d from reported consumption was assessed for all dogs in ideal body condition based on manufacturers’ or US Department of Agriculture database information. To assess the respective parameters across competition levels (novice, open, master/elite), non-parametric or parametric ANOVA or χ^2^ was used to determine significance. There were 494 respondents with usable data. Results showed that approximately 99 % of respondents used treats and 62 % utilised supplements. Of the respondents, 61 % fed primarily commercial dry food. Approximately 25 % of owners fed foods other than commercial dry (i.e. raw/home-prepared or freeze-dried). This 25 % of non-traditional diets included: 11 % home-prepared raw/cooked diets, 11 % commercial raw/cooked diets, and the remaining 3 % were fed commercial freeze-dried raw products. The remaining 14 % fed a mix of commercial dry food and raw/home-cooked blend. Average BCS was 4·7 (sd 1·1). Mean energy consumption of 238 dogs (BCS 4–5/9) was 444 (sd 138) kJ/kg body weight^0·75^ per d (106 (sd 33) kcal/kg body weight^0·75^ per d), with no significant differences observed between dogs at different levels of competition. The mean percentage of energy from treats was 15·1 (sd 12·7) % of overall energy consumption.

Canine agility has become a leading competitive dog sport since its introduction to North America in 1986, with over 40 000 registered members reported in the United States Dog Agility Association (USDAA)^(^^1^^)^. The sport entails a dog working with its handler to navigate thirteen to twenty or more obstacles including jumps, weave poles, tunnels, and contact obstacles in a specific order, under a specified time. These activities require extensive specific training, strength and cross-training activities. This variety and unknown duration of training makes it difficult to assess the energy requirements of dogs participating in this sport.

At this time, to the authors’ knowledge, information on the specific energy requirements of agility dogs, their feeding trends, or supplement use, and ideas surrounding frequency of training and conditioning, and trials per year attended for the average agility competitor across the three different classes typically entered in agility trials (novice, open and master/elite) are unknown. In addition, feeding trends based on form of food (dry, wet or raw/home-prepared), treats provided, energy from treats, and supplement use may be different from those of normal populations. For example, the most recent and comprehensive owner-related study of feeding behaviour claims that over 95 % of owners feed a commercial dry or wet dog food as the primary energy intake, while 96 % of ‘pet dogs’ are provided treats or table foods, often equating to approximately 20 % of their energy coming from treats or table foods, suggesting potential unbalanced and incomplete feeding practices in pet populations^(^^2^^,^^3^^)^. Considering author-perceived (G. K. D., G. M. V., J. J. W.) increasing trends of raw and home-prepared diet feeding becoming more popular among agility dog owners and the propensity to provide large numbers of training treats, a descriptive analysis of feeding practices amongst agility dog owners in the greater Northeastern and Midwestern USA was performed. Our initial aim was to obtain information regarding dog demographics (age, sex and spay neuter status), body condition score (BCS), meal feeding (commercial, raw, home-prepared), treats, and supplement trends, as well as basic training, conditioning and trials attended across the three levels of competition (beginner, amateur, or master in agility competitors). A second aim was to understand the supplement use in agility dogs by assessing all supplements and their categorisation (e.g. joint supplements). The last aim was to better understand the total energy consumption, treat energy consumption and supplement energy consumption patterns of dogs in ideal body condition (BCS 4–5/9) through energy calculations based on manufacturers’ reported metabolisable energy of food, treats and supplements across the three levels of competition.

## Materials and methods

Data were collected for the present study by ‘in-person’ administration of a survey by two of the authors using online Survey Monkey software (SurveyMonkey Inc.). The survey questions were validated for comprehension by mock interviews of ten individual dog owners involved in agility not participating in the study. Answers to all questions were owner reported with the exception of BCS, which was determined by the individual administering the survey. The two individuals administering the survey would score several dogs together per agility event attended to ensure agreement in their evaluations.

The survey was administered at eighteen agility trials over a 10-month period in 2015 in the following states: CT, NY, RI, NJ, NH, OH, IL and PA. Trials included events by the USDAA, American Kennel Club, UK Agility International, North American Dog Agility Counsel and Canine Performance Events to ensure that participants from multiple major US agility organisations were covered. Two USDAA regional championships were included to increase inclusion of participants from a wider geographic area.

Energy intake was calculated based on owner-reported estimations of amounts fed per d of meals, treats and supplements. For commercial diets and treats, energy intake was calculated from owner-reported amounts, and energy content as reported based on manufacturer's reports found either on their website or through telephone contact with the company. Home-prepared or raw diets were calculated from owner-reported amounts and reported energy information on the US Department of Agriculture Food Composition website^(^^4^^)^. To obtain metabolisable energy intake for dogs with ideal body condition, calculations were only performed for dogs with a BCS of 4 or 5 (out of a total score of 9).

Information such as age, sex, spay neuter status and class of dog entry (beginner (novice), amateur (open), master) was collected as general demographic data. Supplements were categorised into hydration and electrolyte supplements, joint supplements (separate from fish oil), fish oil, skin and coat supplements, vitamin and mineral health supplements, post-exercise energy supplements, gastrointestinal aids, antioxidant supplements and ‘other’ supplements. Activity information relevant to the number of times the dogs were conditioned (i.e. other exercise activities besides agility work) on a weekly basis, times per week trained for agility specifically and number of agility events attended per year over the past year was collected.

### Descriptive and analytical statistics

All data were examined using descriptive statistics with percentages or ratios. Sex was categorised into male neutered, male intact, female spayed and female intact. Meal feeding practices were placed into one of three categories: (1) commercial food (70 % of diet or more); (2) primarily home-prepared, raw and/or freeze-dried (calculated as 70 % or more); or (3) mix of commercial and home-prepared, raw and/or freeze-dried. Supplement use was reported as percentage of respondents for each supplement group out of the entire population surveyed. BCS categorisation, level of competition (beginner, open, master/elite), mean age, mean owner-reported weight of competitors, top five breed popularity, trials attended per year, bouts of agility training per week and bouts of conditioning exercise per week were provided as a ratio of respondents or percentage. Of the respondents, a subset of dogs with BCS 4 and 5 was further assessed for relative mean energy intake, reported as kcal/kg body weight^0·75^ per d (kJ/kg body weight^0·75^ per d). This is further assessed as percentage of energy from primary food (commercial or home-prepared/raw/freeze-dried) and percentage from treats and supplements. In addition, the percentage of dogs receiving over 20 % of their total energy intake from treats and supplements was calculated.

To better understand how competition level affects these parameters either χ^2^ testing (categorical) or parametric ANOVA was performed to determine differences between the competition levels regarding age, sex, supplement use, type of feeding (commercial, raw/home-prepared/freeze-dried, mix of these), BCS, training sessions per week, conditioning sessions per week, and trials attended per year (Prism 6.0 Software). Depending on normality testing (Shapiro–Wilks), similar parametric or non-parametric ANOVA across competition level was performed utilising data from dogs for total energy intake, energy intake from treats and energy intake from supplements.

## Results

Of the 501 respondents to the survey, 495 had complete data that could be utilised for analysis of all parameters. Seven were discarded due to poor dietary information. Of the 494 respondents, the average age of agility dogs was 6·0 (sd 2·7) years and the average weight was 16·8 (sd 8·6) kg. There were fifty-seven breeds represented in the dataset. The top five breeds were border collie (*n* 107), Shetland sheepdog (*n* 53), Australian shepherd (*n* 30), golden retriever (*n* 28) and cocker spaniel (*n* 14). BCS was assessed on 444 of the 494 respondents (some dogs were not present at the time of the survey), averaging 4·7 (sd 1·1), with no dogs being BCS of 1, one being BCS 2, nineteen being BCS 3, 219 being BCS 4, 123 being BCS 5, fifty-six being BCS 6, fifteen being BCS 7, eight being BCS 8 and three being BCS 9. There were 180 neutered males, sixty-nine intact males, 204 spayed females and forty-one intact females. The average number of agility training sessions was 2·5 (sd 1·8) per week, while the number of conditioning sessions (other athletic endeavours) was 4·8 (sd 2·2) per week and number of competitions per year attended averaged 20·2 (sd 10·2). Feeding patterns showed that 304 (61 %) respondents fed primarily commercial dog food, 122 (25 %) home-prepared, raw and/or freeze-dried and sixty-eight (14 %) fed a mix of commercial and home-prepared, raw and/or freeze-dried. Only one respondent fed canned food primarily. Of the home-prepared, raw and/or freeze-dried feeders, fifty-five fed home-prepared cooked or raw foods (11 %), fifty-two (11 %) fed commercial raw or home-prepared foods and fifteen fed freeze-dried raw (3 %). Of the respondents feeding a mix of raw and kibble, twenty (4 %) fed home-prepared raw or cooked foods, thirty-two (7 %) fed commercial raw foods and sixteen fed freeze-dried raw products (3 %).

Treat consumption was nearly universal, with 488 respondents using treats (99 %). Fewer dogs used supplements (308/494; 62 %). Of the 62 %, 77 % were given joint supplements, 33 % fish oil, 28 % vitamin and minerals, 21 % gastrointestinal support, 18 % herbal, 14 % post-exercise energy/protein, 7 % antioxidant, 6 % other, 3 % immune support, and 1 % skin and coat.

Of the 495 participants, forty-nine dogs were novice competitors, fifty were open competitors and 395 were master/elite competitors. Sex, BCS, type of feed, supplement use, agility training bouts per week and conditioning sessions per week across the three levels of competition showed no significant differences across groups. Age and number of competitions showed differences with increasing age and number of events for the master/elite when compared with the novice and open classes (see Table 1; *P* < 0·001).

**Table 1.** Demographics, feed and competition descriptive statistics and assessment of parameters across all three groups of competition level (Mean values and standard deviations; numbers)

	Novice		Open		Master/elite		
	Mean	sd	Mean	sd	Mean	sd	*P*
Sex (*n*)							
Female	26		27		195		
Male	23		23		204		
Female spayed (*n*)	20		24		162		
Female intact (*n*)	6		3		33		
Male neutered (*n*)	19		17		145		
Male intact (*n*)	4		6		59		0·57
Age (years)	3·9	2·9	4·7	2·0	6·4	2·6	<0·001
Average body condition score	4·6	1·0	5·0	2·0	4·7	1·1	0·38
Commercial fed (*n*)	30		27		247		
Home-prepared/raw fed (*n*)	11		11		100		
Mixed fed (*n*)	8		6		54		0·83
Supplement use (*n*/*N*)	19/49		22/50		263/395		<0·001
Sessions of training (per week)	2·7	2·1	2·9	1·8	2·4	1·8	0·07
Number of trials (per year)	11·5	9·7	14·5	9·4	22·0	9·5	<0·001
Sessions of conditioning (per week)	4·7	2·6	4·9	2·1	4·9	3·6	0·09

In dogs with a BCS of 4 or 5 (total number 346), evaluation of energy intake from meals, treats and supplements was carried out in 238 respondents with complete information. Of the 238 respondents, twenty-nine were novice, seventeen were open and 192 were master/elite class participants. There were no differences in total energy consumption or energy consumption calculated from main meals, treats or supplements across the three competition levels (Table 2). Further assessment of the respondents’ treat consumption revealed that approximately eighty-seven of these respondents (36 %) were feeding over 20 % of the daily energy intake as treats and/or supplements, while this consumption dropped to seventy respondents (29 %) when assessing dogs that were being fed over 20 % of their energy from treats alone.

**Table 2.** Total energy consumption from meals, treats and supplements across the three competition levels (kcal consumption/kg body weight^0·75^ per d)* (Mean values and standard deviations; medians and ranges)

	Novice		Open		Master/elite		
	Mean	sd	Mean	sd	Mean	sd	*P*
Total energy intake	103·7	34·2	100·2	18·1	106·8	33·3	0·63
Meal energy intake	79·4	26·3	85·7	17·7	85·9	32·5	0·82
Treat energy intake							0·18
Median	15·4		8·0		12·1		
Range	2·9–61·8		0–30·6		0–188		
Supplement energy intake							0·08
Median	1·0		0·6		5·6		
Range	0–17·4		0–10·8		0–26·4		

* To convert kcal to kJ, multiply by 4·184.

## Discussion

Feeding trends in dogs across the USA have been examined, suggesting that 98 % of dogs consume primarily commercial dog food, with approximately 3 % of dogs being fed 50 % or more raw or home-prepared foods^(^^3^^)^. Our assessment of a small subset of the general population as competitive agility dogs suggests that 25 % are being fed primarily home-prepared, raw and/or freeze-dried food and this increases to nearly 39 % when we include dogs that are being fed a mix of commercial dry food with at least 30 % of the meal volume coming from home-prepared, raw or freeze-dried foods. This is either a trend in this specific population and/or there has been a significant increase in the number of raw and home-prepared food feeders since the original survey in 2004^(^^3^^)^. Overall this brings to light the possibility of improperly balanced diets, since over 60 % of diets formulated by owners from books, Internet sources and other media outlets show one or more nutrient deficiencies^(^^5^^,^^6^^)^.

When examining agility dogs approximately 99 % of dogs were fed treats, which is similar to the trends seen in pet dogs in the UK^(^^7^^)^. The use of treats in agility training is very popular, and it was evident that based on competition level that treats were used slightly more frequently early in training in novice and open competitors. Agility competition dogs often require treats as positive re-enforcement for good performance, which may lead to excessive treat consumption. Prior studies in adult pet dogs have shown that the average consumption of table foods or treats is approximately 20 % of the average daily energy which might dilute or enhance specific nutrients in the diet, potentially creating imbalance^(^^2^^)^. Over 29 % of respondents were feeding over 20 % of total daily energy consumption from treats; when supplements were added into the calculations for daily energy consumption this increased to over 35 % of respondents.

This type of consumption may not be a problem when dogs are highly active and have higher energy demands as athletes, but evidence from our study suggests that this cohort of agility dogs are not consuming much more than the average pet dog energy requirements to remain at a BCS of 4 or 5 out of 9^(^^8^^,^^9^^)^. This energy intake is lower than reported by other canine athletes or even recent meta-analyses of sedentary or kennelled dog requirements^(^^8^^,^^9^^)^. Factors that may be involved in daily energy requirements include activity, breed, body weight, BCS and husbandry. When examining ‘pet dog’ feeding studies often the energy necessary for maintenance is similar to our findings, and potentially even lower^(^^10^^–^^13^^)^. Considering the propensity for border collies in our population when we compare our findings with those of Butterwick & Hawthorne for low level activity in pet border collies (1–3 h per d of activity), our findings are nearly identical^(^^12^^)^. Another similar study of pet dogs by Sunvold *et al*.^(^^13^^)^ suggests even lower overall energy intake in the average pet dog, and this study represented a large range of sizes with a predominance of mixed breed dogs. It must be pointed out that our study looked at dogs with a BCS of 4 or 5 out of 9, while the previously mentioned studies either did not mention body condition or dogs had an average BCS of 6 of 9, which is not typical of the agility dog. A more recent assessment of energy intake in dogs that took body condition into account as a variable suggests similar energy intake of dogs with a BCS of 5/9, which is very similar to our findings with the average pet dog consuming 98 kcal/kg body weight^0·75^ (410 kJ/kg body weight^0·75^)^(^^10^^)^. A major limitation of the present study is that the precise energy intake is unknown due to this being a survey-based study and not a feeding trial. It has been shown that there is under-reporting of daily diet logs in human subjects, which may cross over into reliability in caregiver reporting^(^^2^^)^. Furthermore, manufacturers’ metabolisable energy information obtained is often based on labelled guaranteed analysis and that fat content of foods is often higher; therefore the reported energy from manufacturers may be slightly low^(^^14^^)^. Recent energy estimates also suggest that fibre content of the diet plays a significant role in metabolisable energy whereby manufacturers’ use of modified Atwater equations will under-predict the energy density of lower-fibre foods, particularly those higher than 3·5 kcal/g (14·6 kJ/g)^(^^8^^)^. Regardless of these limitations, it is likely that agility dogs are still within the energy range typical of ‘pet dogs’ and do not reach the energy requirements of greyhounds, which have been shown to require just above two times the resting energy requirement as their daily energy requirement^(^^11^^)^; however, further studies are warranted to confirm these findings.

### Conclusions

Energy needs for agility dogs in athletic body condition appear to fall within the energy needs of the average household ‘pet’ dog, and there are no differences observed based on the level of competition. Treat consumption in agility dogs shows that approximately one-third of these dogs are getting over 20 % of their daily energy from treats and/or supplements, providing a platform for manufacturers to make more complete treats for this unique group of dogs. Similarly, nearly two-third of agility dogs receive a supplement of some kind, with joint supplements being the most common and supplement use increasing with mastery and/or age of dogs. Feeding trends in these dogs are also atypical, with over 25 % of agility dogs receiving a major proportion if not all of their energy from raw or home-prepared foods rather than commercial extruded pet foods.
